# Supplementary material for: Megalencephalic Leukoencephalopathy with Subcortical Cysts Disease-Linked MLC1 Protein Favors Gap-Junction Intercellular Communication by Regulating Connexin 43 Trafficking in Astrocytes
Source: Cells. 2020 Jun 8;9(6):1425. doi: 10.3390/cells9061425 (PMC7348769; doi:10.3390/cells9061425)
Supplement: Supplementary file 1 [file cells-09-01425-s001.pdf]

Supplementary material

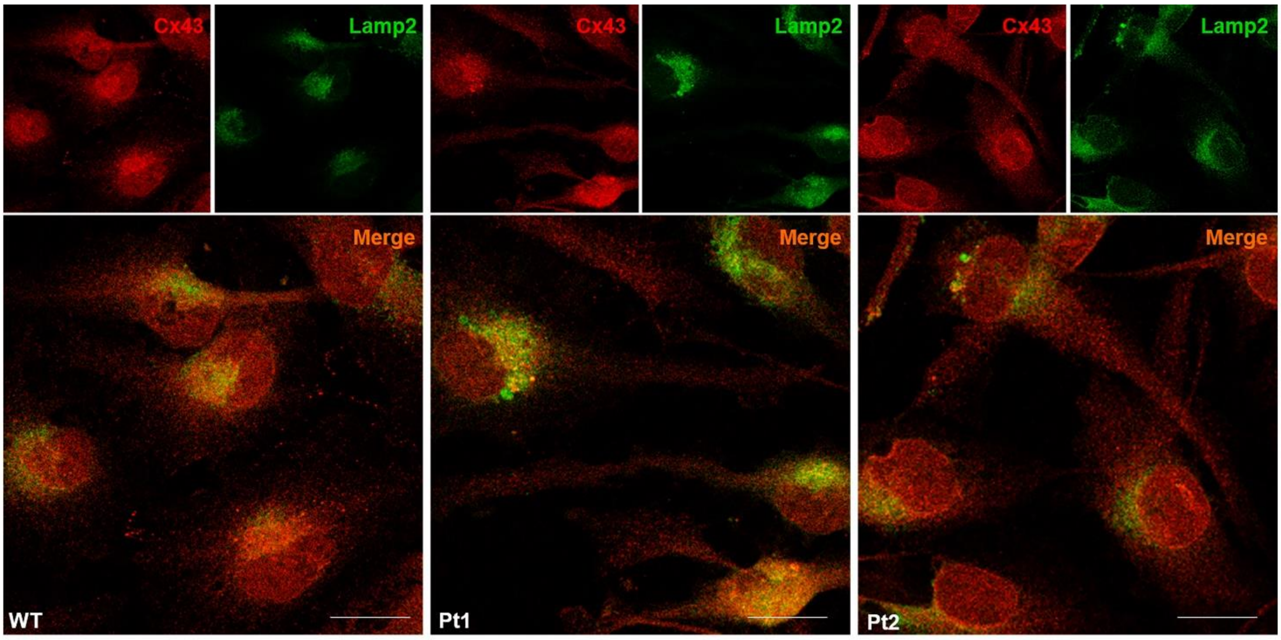

Figure S1. Analysis of Cx43 protein intracellular trafficking in U251 astrocytoma cells overexpressing MLC1 WT or carrying the pathological mutations Pt1 and Pt2. Double immunofluorescence stainings with anti-Cx43 pAb (red) and anti-Lamp2 mAb (green) that recognizes lysosomes shows no significant differences in Cx43/Lamp2 colocalization in all the MLC1 expressing cell lines analyzed. Scale bars = 20 μm.

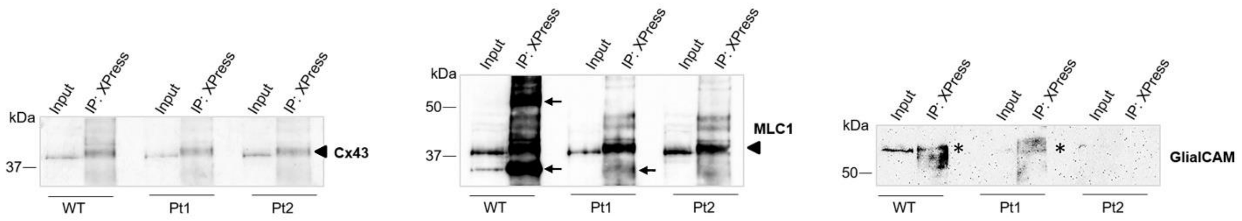

Figure S2. Co-IP assay. Lysates (1.5 mg/ml) derived from U251 cells expressing MLC1 WT or Pt1 and Pt2 mutants were immunoprecipitated under conditions that preserve protein-protein interactions using monoclonal anti-Xpress mAb (1-2 μg/sample) recognizing MLC1 protein, as described in Materials and Methods section. WB analysis of immunocomplexes indicates that the anti-Xpress mAb was able to immunoprecipitate MLC1 and its interactor GlialCAM in WT and, at lower levels, also in Pt1 mutant cells

(arrows and asterisks, respectively). No MLC1 specific bands are detected in Pt2 cells that express a truncated, rapidly degraded form of MLC1, and thus used as negative control. No specific bands are also observed in all the MLC1 expressing cell lines when anti-Cx43 pAb is used. Arrowheads mark unspecific bands recognized by anti-MLC1 and anti-Cx43 pAbs. Input represents the starting material (cellular lysate). For MLC1 protein detection an anti-MLC1 pAb has been used. The molecular weight standards in kDa are indicated on the left.

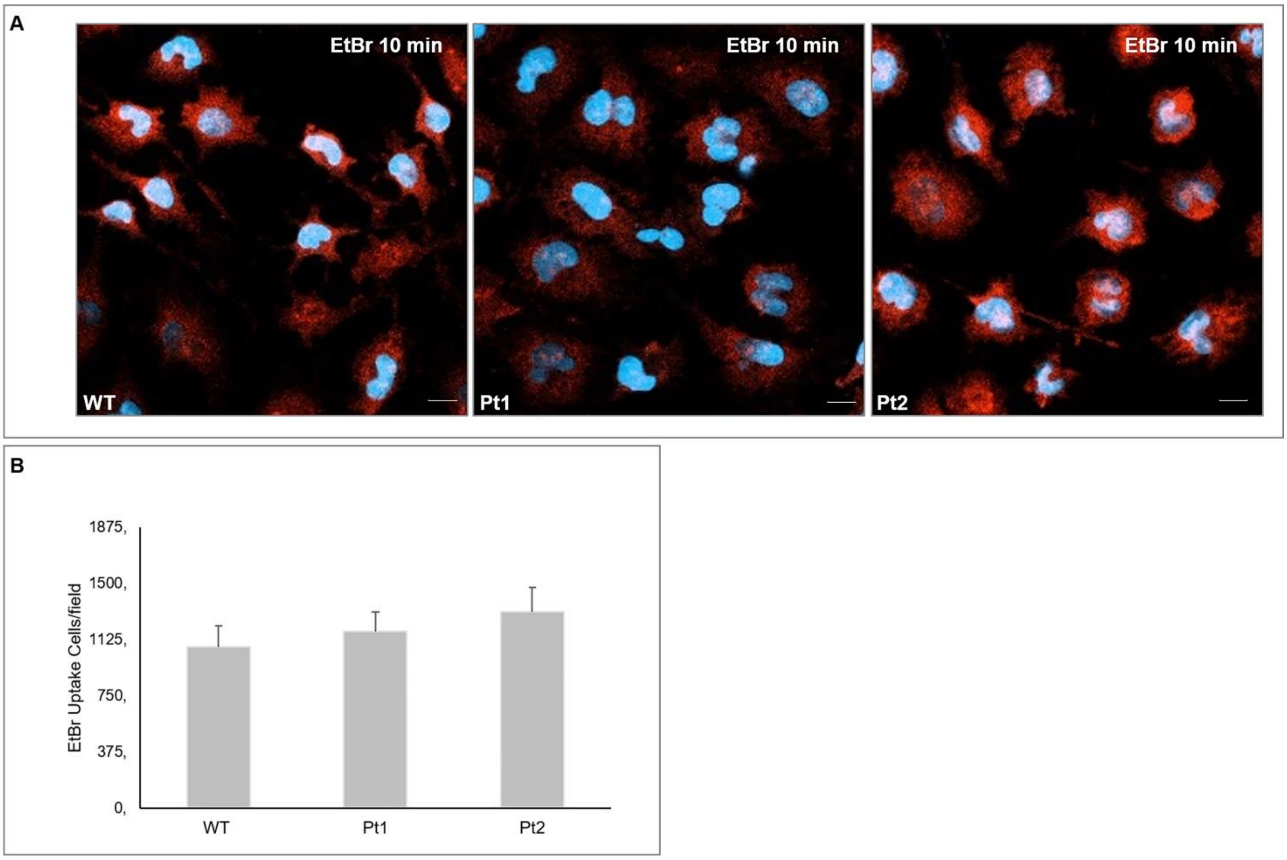

Figure S3. Evaluation of astrocytic hemichannel activity in U251 cells expressing MLC1 WT or carrying Pt1 or Pt2 mutations. (A) Representative images depicting ethidium bromide (EtBr) uptake (red) via hemichannels in the three cell groups after 10 min of EtBr treatment. DAPI staining is used to identify cell nuclei (lightblue). Scale bar = 20 μm. (B) Quantification of EtBr fluorescence intensity by confocal analysis. EtBr mean fluorescence intensity evaluated in single cells shows no significant differences in EtBr uptake among the three cell lines. Three experiments were carried on in triplicate. Statistical analysis was performed by one-way ANOVA.

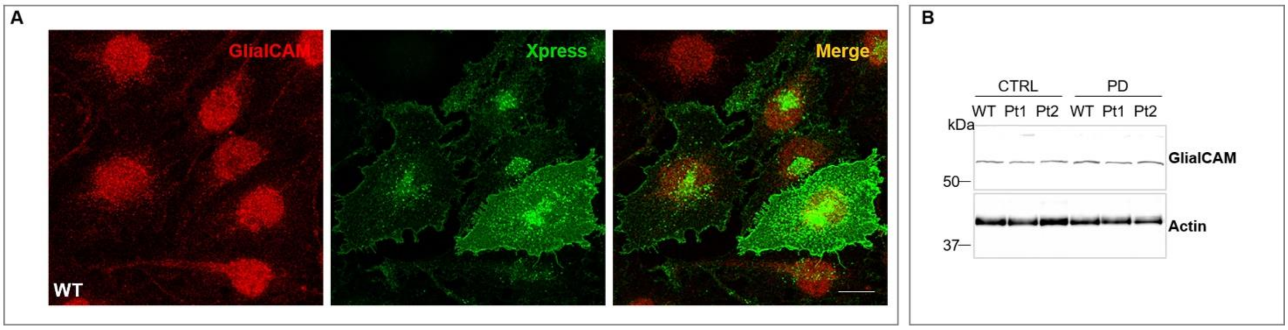

Figure S4. Glialcam protein expression in U251 astrocytoma cells overexpressing MLC1 WT or carrying Pt1 and Pt2 mutations. (A) Double immunofluorescence staining with anti-GlialCAM pAb (red) and anti-XPress mAb (green) to detect MLC1 protein shows that GlialCAM protein is not localized at the plasma membrane but it is retained in the cytoplasm in WT MCL1 expressing cells. (B) WB analysis performed with 40 µg of protein extracts derived from MLC1 expressing cell lines, untreated (CTRL) or treated with 50 µM of the pERK1/2 inhibitor PD98059 (PD) for 1 h shows no differences in GlialCAM expression levels among the cell lines analyzed, in presence or absence of PD. Actin is used as loading control. Molecular weight markers are indicated on the left (kDa). One representative experiment out of three performed is shown.
